# Fatigue as a key human factor in complex sociotechnical systems: Vessel Traffic Services

**DOI:** 10.3389/fpubh.2023.1160971

**Published:** 2023-04-14

**Authors:** Fernando Crestelo Moreno, Verónica Soto-López, Deva Menéndez-Teleña, Joaquín Roca-González, Juan Suardíaz Muro, Cristina Roces, Mercedes Paíno, Imma Fernández, Luis Alfonso Díaz-Secades

**Affiliations:** ^1^Spanish Maritime Search and Rescue Agency, Cartagena, Spain; ^2^Department of Automation, Electrical Engineering and Electronic Technology, Technical University of Cartagena, Cartagena, Spain; ^3^Department of Marine Science and Technology, University of Oviedo, Oviedo, Spain; ^4^Department of Psychology, University of Oviedo, Oviedo, Spain

**Keywords:** complex socio technical systems, fatigue, vessel traffic service, maritime psychology, maritime system safety

## Abstract

**Background:**

Vessel Traffic Services (VTS) are considered a subsystem of people, whose mission is to improve the effectiveness and efficiency of maritime transport within the maritime system. VTS operate as a control system where Vessel Traffic Services Operators (VTSOs) must cope with a complex environment to make up for safe and efficient maritime transport, so it is essential to understand how these operators maintain control through adapting to the uncertain and dynamic environment of maritime traffic. This multidisciplinary study explores how human factors within this complex sociotechnical system, means VTS, play a role in maritime safety, specifically focusing on fatigue, which is considered a key variable for VTSOs’ performance. In this context, the research has focused on the assessment of fatigue with psychological and operational instruments to highlight the importance of the human element in CSSs.

**Objective:**

To study the intra and inter-individual differences in fatigue ratings and their relationships with other personal and environmental variables: mental workload, work shifts, reaction time, and hours of usual sleep.

**Methods:**

The study was conducted in two of the 19 Spanish Maritime Rescue Coordination Centers (MRCCs) with a sample of 23 active VTSOs (82.14% of the staff). Both objective and standardized subjective measures were used to assess fatigue and associated sleepiness (Borg RPE, Nasa-TLX, Stanford Sleepiness Scale, and Self-Assessment Manikin Scale).

**Results:**

A significant positive correlation between fatigue and mental workload was found, being more prevalent in the night shift, which shows a bigger variation in these variables. A significant difference was found in the increase in fatigue experienced throughout the shift depending on the hours of usual sleep, being smaller in the group of subjects reporting to sleep more.

**Conclusion:**

The human element is key to maritime safety related to Vessel Traffic Services, so it is of paramount importance to consider certain measures to counteract the negative effects of fatigue. A proper organization of duties on/off periods, assessment of work and rest schedules, and the implementation of fatigue management programs based on sleep education are recommended.

## 1. Introduction

Maritime shipping has been the main means of transport and trade since its inception in the 6th millennium BC (1). Since then, there has been an increase in both the number and size of ships, which, in turn, has resulted in increased congestion and complexity of maritime traffic. Nowadays, maritime transportation handles 90% of the world’s goods (2) and, according to the Baltic and International Shipping Council (BIMCO), by the end of 2025, the fleet is expected to have a Compound Annual Growth Rate (CAGR) of 1.25% with a global fleet growth by 6.4% (3).

During the WWII, the Vessel Traffic Services (VTS) arose as a subsystem, within the maritime system, to improve the effectiveness and efficiency of maritime transport. These VTS are internationally recognized as a navigational safety measure through the International Convention for the Safety of Life at Sea 74/78 (SOLAS).

Vessel Traffic Services are defined as shore-based services, implemented by Governments with responsibilities for their adjacent maritime areas, with the ability to interact with maritime traffic and to respond to changing circumstances within a vessel traffic service area, in order to enhance the safety and efficiency of navigation, contribute to the safety of life at sea, and support the protection of the environment (4).

In this context, SOLAS Contracting Governments must provide the establishment of VTS when, in their judgment, the volume of traffic or degree of risk justifies such services. Nevertheless, VTS can only be mandatory in maritime areas located in the territorial seas of a coastal state (5).

In recent years, the maritime industry has seen an increasing integration in both technology and automated systems, and faces big changes over the next decade, when technological development opens up new ways of operating vessels: autonomous ships, automatic route plans exchange, satellite ship monitoring, etc. This is why Vessel Traffic Services are considered sociotechnical systems resulting from a combination of technological systems, human interfaces, and human-intensive organizational systems.

A complex sociotechnical system, hereinafter CSS, which is a term coined by Tavistock Institute in the 1950s, is a type of system that combines both social and technical components. It can it can be defined as:

“…increasingly common classes of large-scale system that feature a combination of technological systems (where hardware and software technology feature as significant elements within the system), human interfaces, and human-intensive organizational systems” (6).

These systems are found in many different areas, including healthcare, education, transportation, and business. On the one hand, the social components of a CSS include people, organizations, regulations, and policies that interact with the system. On the other hand, the technical components consist of hardware, software, and networks that are used to enable the system to function. Therefore, CSSs are designed to be adaptive and dynamic, allowing for changes to be made to the system as needed. This is important because it allows the system to respond to changing contexts, user needs, and regulations. For example, a maritime system may need to be updated to comply with changing regulations or to meet the needs of an increasingly diverse maritime traffic as maritime autonomous surface ships (MASS).

**Figure 1:**
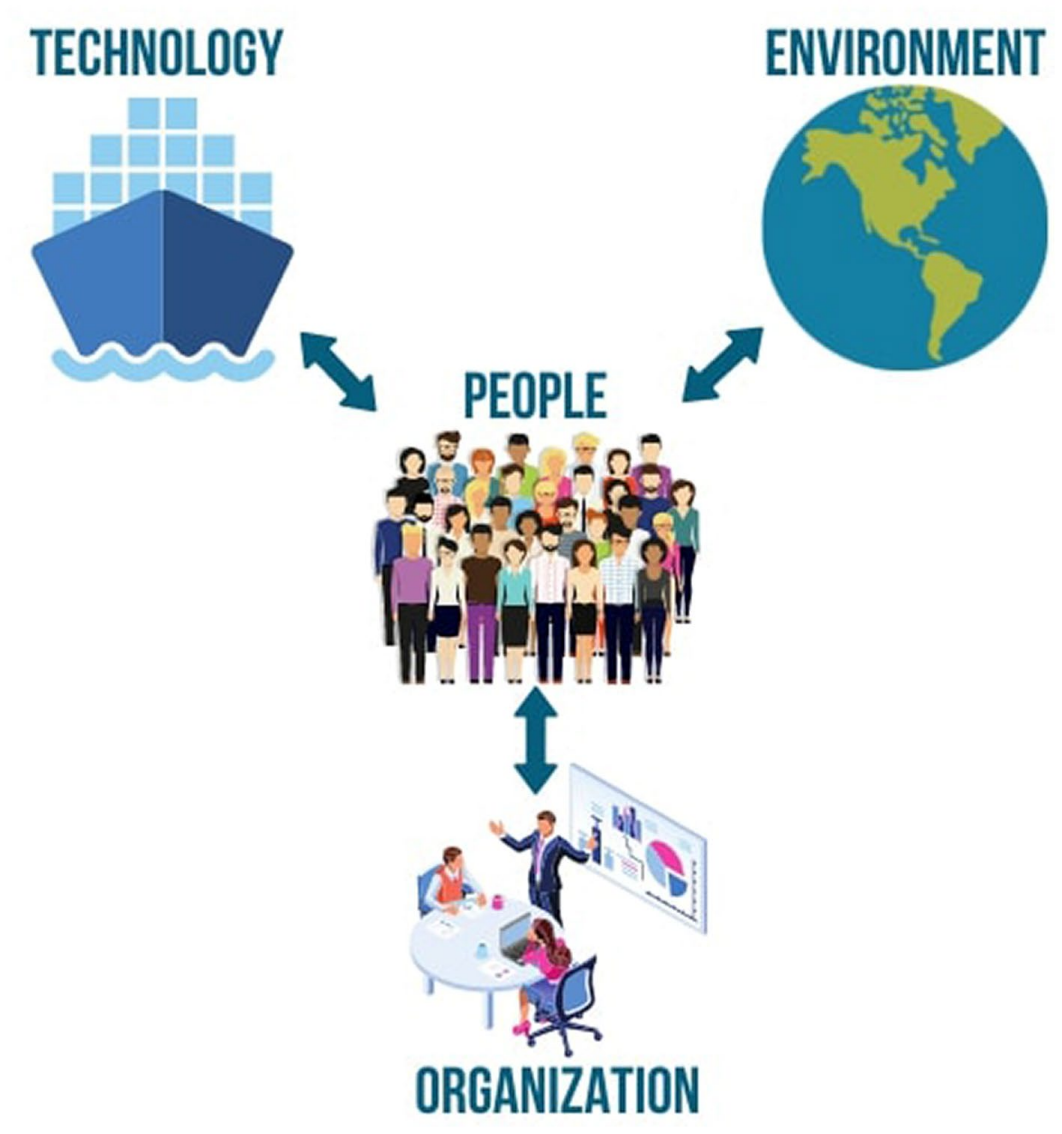
Maritime System Is a CSS [adapted from Rothblum (7)].

In summary, a complex system is one that has more information than that provided by each of the parts that form it, so the maritime system is a complex system of people interacting with technology, the environment, and organizational factors (Figure 1).

VTS can be described as a CSS, where hardware and software technologies are presented as significant elements within the system. VTSOs are part of this system of people, within the maritime system, and they are in charge of controlling and managing maritime traffic through the use of decision support tools (DST), which are based on technological improvements.

The maritime scenario, in turn, is a complex system in constant evolution due to factors such as the increase in the volume and size of ships or the modernization of the sector itself. The introduction of new technologies (Electronic Chart Displays (ECDIS), Automatic Identification Systems (AIS), Marine Autonomous Surface Vessels (MASS), etc.), has changed the image of the classical seafarer seen as *sea dog*, to a more technical profile.

The VTS is, also, a changing CSS (8, 9), in which it is essential to understand how the system maintains control by adapting to the uncertain and dynamic environment that constitutes maritime traffic.

VTSOs are crucial to keep the VTS system operational in the face of different disturbances or deviations suffered by the operators themselves: fatigue, workload, teamwork, communications, resilience, etc., derived from daily operations between the shore system and the vessel system (10).

If the environment changes, humans can, sometimes, self-regulate to moderate risk and to continue within their own established limits of risk tolerance (11). To maintain an equilibrium to any change in the external system, the VTSOs will require a change in their internal system to try to keep their internal variables within the right limits. These reactions are called homeostatic reactions (12). A clear example is the self-regulation of the internal temperature of our organism, which can regulate itself around 37.4°C (99.32°F), although the external temperatures can vary by up to 40°C (104°F) (13). This type of regulation is inevitable and automatic, although other types of regulation can be conscious and deliberate or subconscious and automatic (14).

But, as human fatigue has been identified as the main cause of accidents in the maritime sector (15–17), it means that operators cannot always self-regulate this variable by themselves; consequently, measuring fatigue and its correlates becomes crucial for establishing means to regulate it and for the right functioning of the entire system.

At this point, it is important to introduce the concept of operational fatigue, as defined in the context of the AMES Research Center and NASA projects (18), which involves a set of manifestations in the user’s state associated with the loss of physical and cognitive resources throughout task performance. Increased fatigue is associated with decreased vigilance being sleepiness one of the factors contributing to the onset of fatigue.

Fatigue, therefore, leads to a reduction in both physiological and psychological abilities. It can come out in different ways, depending on the characteristics of the task being performed (e.g., a firefighter shows different changes from a surgeon’s, a pilot’s, or a vessel traffic service operator’s, due to the different psychophysiological resource demands of each of these professions) (19).

Due to the importance of fatigue in the maritime sector, this research is focused on the assessment of the fatigue through a set of objective (an application) and subjective (questionnaires) instruments to highlight the importance of the human element in CSS.

## 2. Bibliometric analysis

In order to get a visual picture of the state of the art, a bibliometric analysis was conducted doing a search in Scopus of the keywords related to the area of study (maritime & safety & fatigue & vessel traffic or services or operator or human element or human factor or complex systems or safety management or behavior or human error or human-machine).

The final search delivered 1,516 documents that were analyzed with the software used for constructing and visualizing bibliometric networks “VosViewer.” Two hundred and twenty-eight critical (10 or more appearances) keywords addressing Complex Systems in VTSOs were found. The most recurrent items are human (309 occurrences), ships (196 occurrences), and fatigue (155 occurrences), which reinforces the importance attach to “fatigue” in this occupational area.

**Figure 2:**
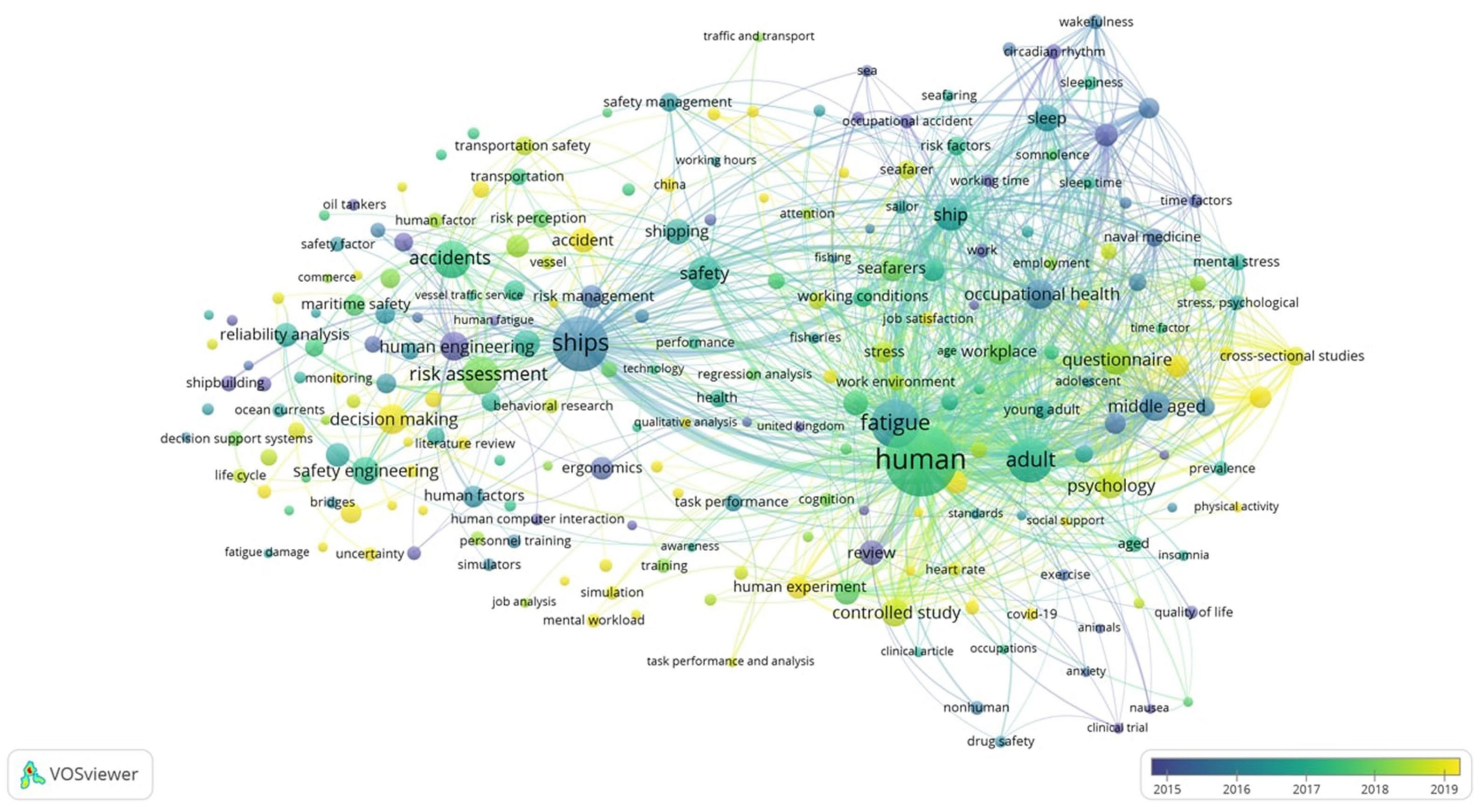
Overlay visualization of co-occurrence analysis from 2014 to 2022 (Source VosViewer).

The following Figure 2 shows a co-occurrence keywords analysis. It represents the evolution on the research topics from 2014 to 2022. The most mentioned topics have been changing along this period. In 2014–2015, the focus of studies was on human engineering, ergonomics, and human factor among others. In 2015 ships, somnolence, fatigue, and occupational health appear as the most used keywords. Around 2017, the focus is on human, adult, accidents, and safety engineering. In more recent years, the center of attention has pivoted into psychology, questionnaire, accidents, and controlled and cross-sectional studies that will help the decision-making process.

## 3. Materials and methods

### 3.1. Study sample and location

**Figure 3:**
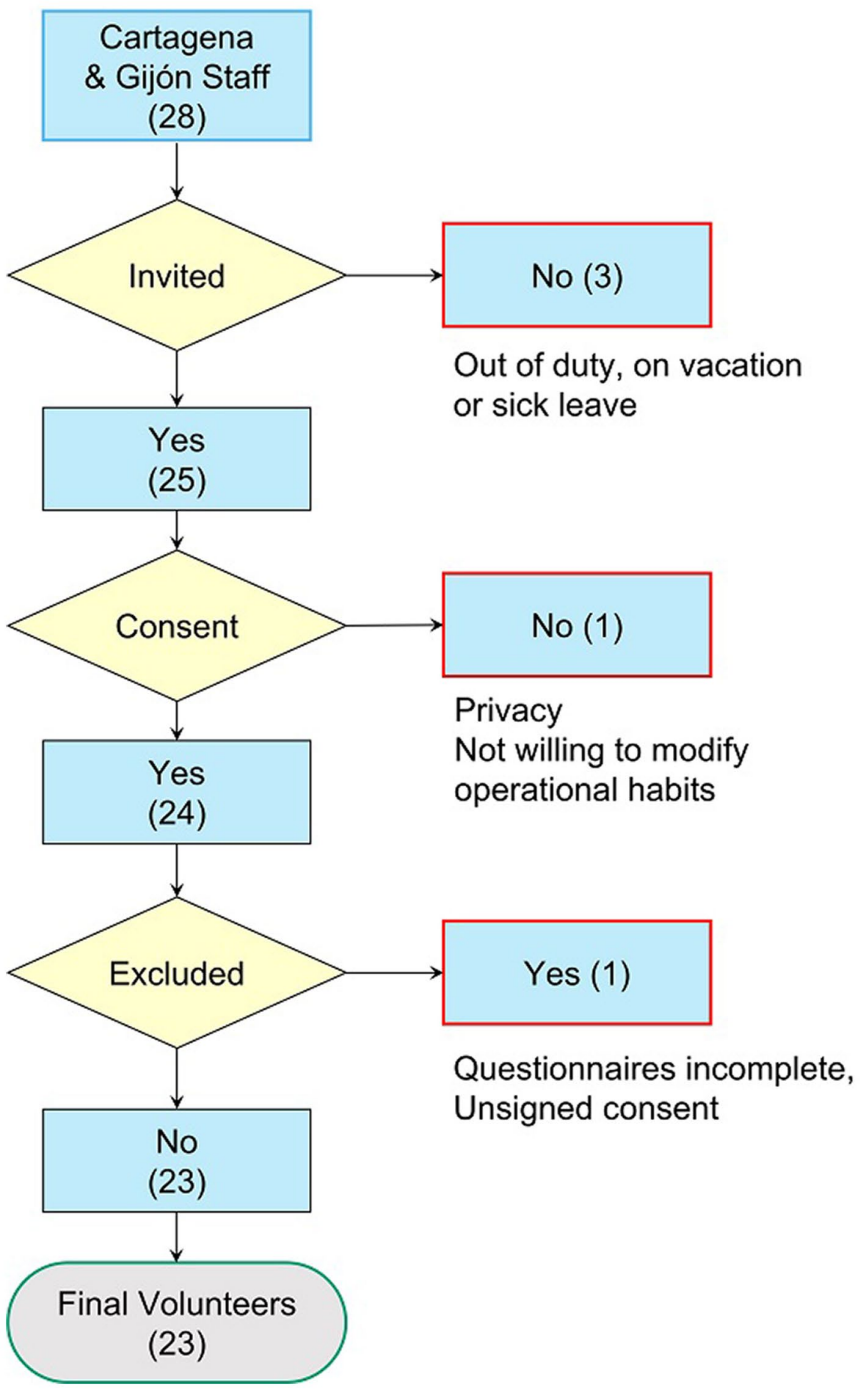
Flowchart for study recruitment.

The study was conducted in two of the 19 Spanish Maritime Rescue Coordination Centers (MRCCs) with a sample of 23 active VTSOs out of a possible 28 that would correspond to the total staff of these centers, which represents a local participation rate of 82.14%. The remaining 17.86% did not participate in the survey for a variety of reasons, including holidays, sick leave, privacy, or incomplete questionnaires (Figure 3).

In global terms, considering that Spanish VTSOs staffing at the time of the study was close to 300, this represents a sample of 7.66%.

The first center is located in Cartagena and the second in Gijón. These centers were chosen for their unique and antagonistic characteristics, both geographically, on opposite sides of the country, as well as climatic conditions, workload, number of emergencies attended to, or VTSOs’ experience. Both of the centers have two operators per watch, plus the reserves, making a total of 14 VTSOs in each center to cover 24/7/365, seen to their duties. The shift organization is carried out in order to guarantee 24-h service coverage, with the following schedules: 7-h watches in mornings and afternoons, and 10-h at night (Table 1).

**Table 1:** VTSOs schedules.

Shift	Schedule
Morning	08.00–15.00
Afternoon	15.00–22.00
Evening	22:00–8:00

Three days off for every three shifts in a row.

This study recruited participants of a wide range of age, work experience, or gender [*N* = 23 mean (±SD) age = 42 ± 9.56 yrs.; total experience = 7.74 yrs.; 60.87% male]. All the participants had nautical training, as this is mandatory for Spanish VTSOs.

In order not to bias the results, since some work shifts are more unfavorable than others on sleepiness, due to circadian rhythms, volunteers were selected by simple random sampling to reduce this bias to a minimum and create a sample that fully represents the study population.

In this way, and taking into account that the on-call schedule is established in advance by the center manager, this selection process was based on a lottery system, assigning a number to each VTSO, choosing the volunteer daily through a number generator software, discarding the numbers already drawn.

The design and procedures of the study were previously reviewed and approved by the Scientific and Ethics in Research Committee of Technical University of Cartagena, and signed informed consent forms were requested to all of the participants prior to their inclusion as research subjects. Participants were free to withdraw from the study at any time.

### 3.2. Materials

Materials used in this study included an *ad hoc* developed Task Attention Control software (TAC), and standardized self-administered fatigue and sleepiness questionnaires and rating scales (Borg RPE, Nasa-TLX, Stanford Sleepiness Scale, and Self-Assessment Manikin Scale).

#### 3.2.1. Task attention control software

TAC is a mobile application programmed in JavaScript, functional from Android 4.4, whose purpose is to obtain an objective temporary measurement of the participant’s performance in a task that consists of clicking a button at the sound of an alarm. For this, a user interface with a functional and minimalist design has been developed, this implies a neutral background and no drawings that can distract the participant.

The use of the TAC is very simple: once the application is started, a red shutdown button will appear on the screen, which will turn green once it has been pressed, thus starting a counter, not visible on the screen. Once the programmed time has been reached, which has been stipulated at 15 min ± x random seconds to avoid predictability, an alarm is triggered, at which point the software counts in milliseconds (visible on the screen) the reaction time of the participant’s acknowledgment of the alarm, generating an internal file with this data. Then, the reaction times disappear from the screen, the button turns green and the 15 min counter starts again, repeating the cycle until the “stop” option is pressed.

**Figure 4:**
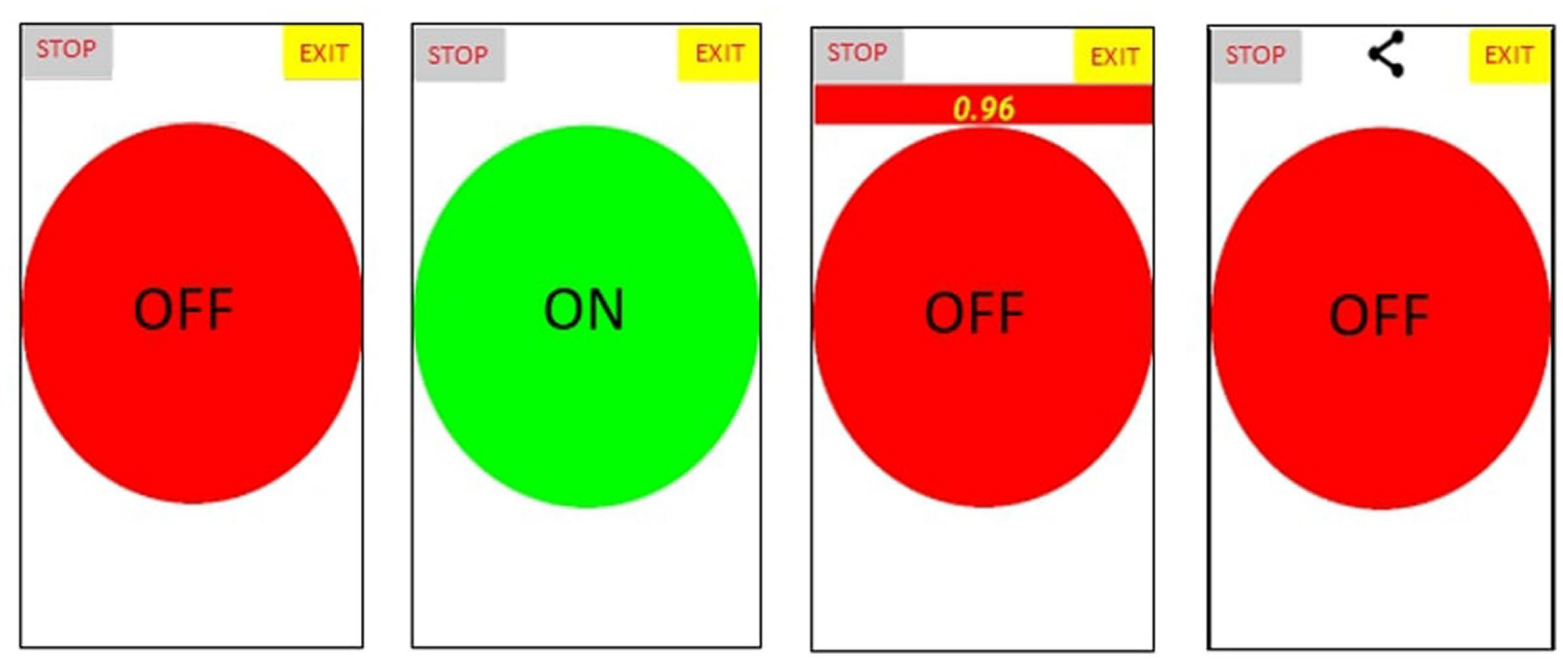
Task Attention Control (TAC) frontend (from left to right: 1 – Initial status; 2 – Running 15 min counter; 3 – Running acoustic alarm; 4 – Stopped and data export).

To finish the task, the *stop* option has to be clicked, and the program gives the option to export a text file (Dropbox, Bluetooth, email, etc.) with all the reaction time records of each participant (Figure 4).

Finally, the *exit* option closes the application.

**Graph 1:**
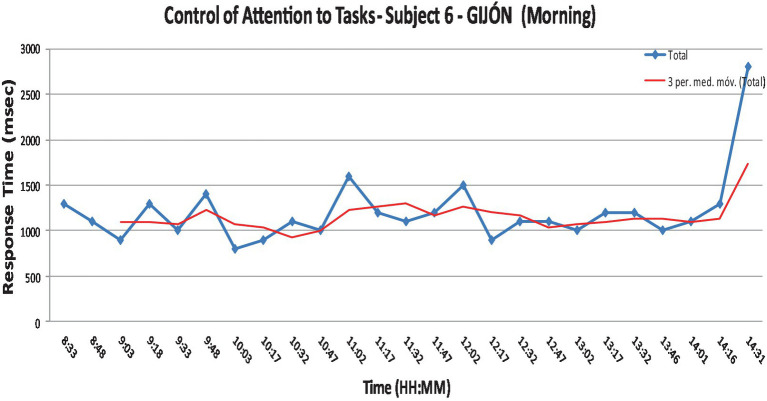
Task attention control sample graph.

The following graph (Graph 1) represents in blue the reaction times (msec) for a given participant. The red line represents the normalization of these times, excluding outliers, caused by reasons external to the response time, due to real conditions work and operator duties.

#### 3.2.2. Instruments

Fatigue questionnaires and sleepiness rating scales are recognized as useful tools to help make a quick and accurate assessment on these issues (20–22). The following paragraphs describe the self-administered paper-and-pencil questionnaires and rating scales used in this study.

##### 3.2.2.1. Stanford sleepiness scale

The SSS is a widely used tool, mainly in research settings, for the subjective assessment of sleepiness. Developed by William C. Dement et al. (23), it is a single-item scale with a Likert 7-point to quantify respondents’ sleepiness levels at a specific point in time, the moment in which the questionnaire is answered; it is, therefore, appropriate for repeated use during a study. It can predict performance on tasks related to alertness (e.g., reaction time, vigilance tests) following total sleep deprivation, not being so sensitive to partial sleep deprivation (24).

##### 3.2.2.2. Borg rating of perceived exertion scale

The RPE scale measures an individual’s effort and exertion, breathlessness and fatigue during physical work, and is relevant for occupational health and safety practice. Developed by the Swedish researcher Gunnar Borg (25), the original version is ranged from 6 to 20 and it has a high correlation to heart rate and with other fatigue related factors (26–29).

##### 3.2.2.3. Self-assessment manikin

The SAM is a non-verbal pictorial assessment tool that measures the pleasure, arousal, and dominance associated with a person’s affective reaction to a wide variety of stimuli. Developed by Bradley and Lang (30), it consists of three single-item scales measuring: response valence/pleasure (from positive to negative), perceived arousal (from high to low), and perceptions of dominance/control (from low to high). SAM ratings can be used to plot directly any object or event into a 2-dimensional “affective space” (31). In our study, only the primary dimensions of pleasure and arousal were used, because they explain most of the variation in emotional judgments.

##### 3.2.2.4. NASA-TLX (task load index)

It is a subjective instrument for the assessment of mental workload (MWL) while the participant performs a task. Developed by NASA Ames Research Center (ARC) (32), it is a multi-dimensional scale that rates performance across six dimensions to determine an overall workload rating. For this study, only the mental demand scale was used, as communications and making decisions are the most frequent activities done by the VTSO’s in duty. Both are recognized triggers of high mental demand and, consequently, fatigue (33–35).

NASA TLX has been successfully used around the world to assess workload in various environments such as aircraft cockpits, command, control, and communication workstations, supervisory and process control, and simulations and laboratory tests. Numerous studies support the reliability and validity of this test (36, 37).

A summary table of the instruments used is shown below (Table 2).

**Table 2:** Scales used to assess sleepiness and fatigue.

	Stanford sleepiness scale	Borg rating scale of perceived exertion	Self-assessment manikin	NASA task load index (NASA TLX)
Type	Self-report	Self-report	Self-report	Self-report
Purpose	Assesses *sleepiness* at specific points in time.	Assessment to measure a person’s *perception* of their effort and exertion, breathlessness, and *fatigue* during physical work.	*Emotional* assessment of the pleasure, arousal, and dominance associated in response to an object or event.	Evaluate *mental workload* demand on an individual performing a specific task.
Items	1	15	3 dimensions pencil-and-paper version with 9-point rating scale per dimension.	Six subscales Mental demand Physical demand Temporal demand Effort Performance Frustration
Limiting values	Valid for comparison purposes.	Cutoff score of 20. Appropriate for any individual Capable of exercising.	Ranged from −4 to +4, with 0 representing the center segment of the scale.	Scale is essentially a line ranging from low (1) to high (20) with 21 marks
Validation reference	Hoddes et al. (43)	Borg (1998)	Bradley and Lang (30)	Hart and Staveland (32) Hart (36)
Target Sample Age	Adults >18	Teenagers 13–18 Adults >18	Both children and adults	Adults >18
Upsides	Very short, only one item. More suitable for repeated use over time for research or treatments.	Yields useful information about fatigue during an activity.	It is brief and effective for measuring emotional responses in a variety of situations.	Provides a quick and simple estimate of operator mental workload. Generic subscales allow the technique to be used across multiple domains.
Downsides	Only assesses sleepiness at a given time.	Lacks established test–retest reliability and validity of physiological measures.	Measures only key dimensions of emotional response	Users of this tool need some training or experience in its use. Laborious and time consuming to conduct the subscale weighing.

### 3.3. Procedure

The study was conducted with a specific group of highly specialized workers, VTSOs, in a real working environment. The procedure was developed on the basis of the specifications extracted from the scientific literature (20, 32, 38–40) and the contributions of three researchers from the Department of Psychology of the University of Oviedo and two experts from the Department of Electronic Technology of the Polytechnic University of Cartagena.

For this research, we collected both subjective and objective data related to fatigue, as one of the main conditioning factors in the efficient operation of a complex system such as the Vessel Traffic Services.

On the one hand, psychological subjective data were obtained with self-report scales and questionnaires, aimed at quantifying fatigue and associated sleepiness (Borg RPE, SSS, SAM, and NASA-TLX).

On the other hand, objective data were collected by measuring the reaction time to a programmed event (acoustic alarm) with the mobile application (TAC) described above to study the impact that sleep loss causes on performance during sustained work (41).

During the data acquisition process, the main researcher is on-site during the VTSOs’ watches, recording all the situational variables in a researcher’s logbook, in order to be able to identify and explain any invalid data due to an anomalous situation.

At the beginning of the procedure, before each shift, the volunteers receive individualized information and assessment about the research, and they are given a consent form, which must be fill in and signed in order to participate. They also fill in a personal information form to make up a demographic database.

At this point, the sleepiness and fatigue tests are given and the TAC software is started at the same time. Participants have to complete these tests before being on duty (previous state). Subsequently, once the shift has started, volunteers are asked to fill in their current status every hour in the same way. Participants are also asked to respond to the TAC alarm each time it sounds, so that their reaction time is recorded.

Finally, the post-tests must be completed at the end of each shift. All tests are collected and archived by the main researcher, who is also the data acquisition manager and responsible for data analysis. All data collected are stored anonymously and each participant is assigned a unique code that is used to identify all files relating to the same subject. The code/participant identification is recorded in a physical paper record and kept under lock and key by the data acquisition manager.

## 4. Analysis

Statistical analyses were carried out using the JASP (version 0.14.1; 42) package. First, a descriptive data analysis was performed of the fatigue values for the SSS and the Borg tests, and on mental load values in the NASA to find out the means and standard deviations for these variables both before and after each of the work shifts (pre and post-work). Additionally, a correlation analysis was carried out to define the associations between fatigue, mental work load and reaction time. To compare the means between two groups, a T-Student was performed. When we needed to compare more than two groups, a univariate analysis of variance (ANOVA) was the choice. To analyze the results, we used the value change of fatigue as a dependent variable, calculated as the difference between pre- and post-work fatigue data from the SSS and the Borg questionnaires. The same was calculated from the NASA questionnaire data to obtain the value change of mental load to use in the corresponding analyses as a dependent variable.

## 5. Results

Twenty-three participants completed the study, most of them during just one shift (18, 78.26%), the rest in different shifts, thus, the total of shifts studied is 32 (50% corresponding to morning from 8:00 to 15:00-, 31.25% evening from 15:00 to 22:00, and 18.75% night shifts from 22:00 to 8:00).

The descriptive statistics for the fatigue and the mental load ratings, split by shifts, for pre-work and post-work are shown in Table 3. There was a considerable variation across individuals, as can be seen in the high standard deviation values.

**Table 3:** Descriptive statistics fatigue and mental load pre and post work.

	M. pre	M. post	E. pre	E. post	N. pre	N. post
Fatigue (SSS) Mean Fatigue (SSS) St. D	1.75 (0.68)	2.13 (0.81)	2.30 (0.95)	2.60 (1.17)	1.67 (0.52)	4.00 (2.10)
Fatigue (Borg) Mean Fatigue (Borg) St. D	8.13 (1.67)	9.38 (1.71)	9.40 (2.12)	10.40 (1.90)	7.33 (1.86)	12.68 (4.50)
Mental Load (NASA) Mean Mental Load (NASA)St. D	20.06 (23.18)	31.56 (22.41)	21.50 (20.56)	39.50 (22.54)	14.17 (10.68)	34.17 (16.56)

M. Pre, Morning shift pre-work. M. Post, Morning shift post work. E. Pre, Evening shift pre-work. E. Post, Evening shift post work. N. Pre, Night shift pre-work. N. Post, Night shift post work. St. D, Standard deviation.

**Graph 2:**
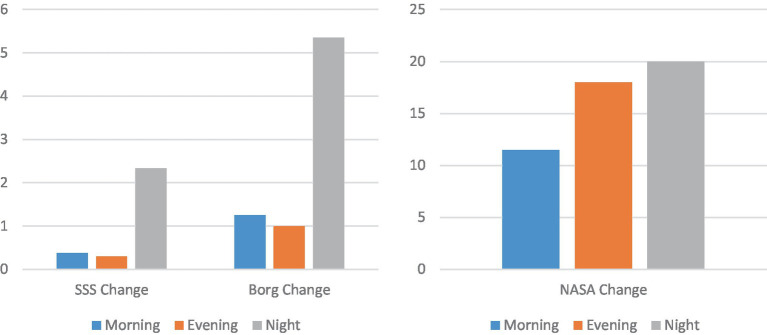
Changes in fatigue (left) and mental load (right) according to the shift.

A bigger increase in fatigue and mental load is associated to the night shift (Graph 2). This association is especially clear for the fatigue values (SSS ANOVA values: *F* = 6.19, *p* = 0.006, eta squared = 0.3) (Borg ANOVA values: *F* = 6.59, *p* = 0.004, eta squared = 0.31) (NASA ANOVA values: *F* = 0.67, *p* = 0.52, eta squared = 0.04).

Spearman’s correlation analyses show significant positive correlations between fatigue and mental work load values (SSS-NASA: *r* = 0.42, *p* < 0.001), (SSS-Borg: *r* = 0.72, *p* < 0.001), (Borg-NASA: *r* = 0.45, *p* < 0.001). No significant links were found between any of these variables and the reaction time. We performed new analyses splitting the group of subjects in two, attending to their work center (Cartagena/Gijón) and the correlations were also non-significant. The small size of the split sample could account for this lack of significance. Furthermore, we studied the differences in reaction time pre and post-work and, although still no significant, a clear tendency was found in the Gijón sample: the measures of the reaction time were higher post work, with an increase of between 100 and 1,500 ms in all the participants, with the exception of one, who had exactly the same value in both instances. The Cartagena sample showed no clear patterns, with some increases, some decreases and some equal reaction times in pre and post measures.

**Graph 3:**
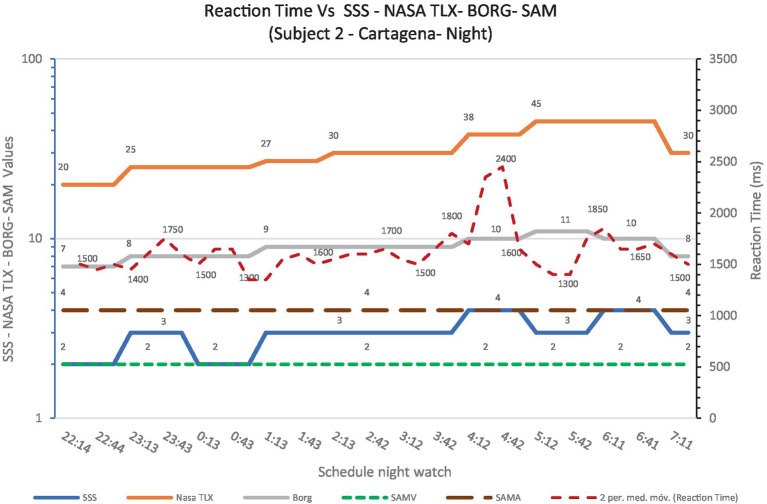
Reaction time Vs SSS – NASA TLX- BORG- SAM (Subject 2 – Cartagena CCS- Night Watch).

The following graph (Graph 3) is a representative snapshot of the night shift. This graph shows that the subject used as an example displays an increase in mental demand (NASA-TLX) as the clock progresses, a slight rise in physical fatigue (Borg) and no increase for the subjective assessment of sleepiness (SSS). It should be noted that, at the end of the shift, the curve values tend to decrease in all volunteers, due to what is called the *spur* effect. This effect is based on the self-balancing of the operators themselves, self-activating, and updating the traffic situation (situational awareness) to transfer the handover.

To study the importance of the subjects’ usual number of hours of sleep, the original sample was divided into two groups: those subjects who reported sleeping fewer than 8 h and those reporting 8 or more hours of usual sleep. As we can see in Table 4, the increase in fatigue experienced throughout the shift is smaller in the group of subjects reporting more hours of usual sleep (SSS T-Student values: *t* = 1.91, *p* = 0.07, Cohen’s *d* = 0.69) (Borg T-Student values: *t* = 1.64, *p* = 0.11, Cohen’s *d* = 0.59); this effect is not significant for the mental load (NASA T-Student values: *t* = 0,69, *p* = 0.5, Cohen’s *d* = 0.25).

**Table 4:** Descriptive statistics change in fatigue and mental load with hours of usual sleep.

	SSS change		Borg change		NASA change	
	1	2	1	2	1	2
Mean	1.105	0.154	2.632	0.923	16.947	12.462
St. D	1.524	1.144	3.353	2.019	16.920	19.590

Group 1 h of usual sleep <8 h (*N* = 19); Group 2 h of usual sleep ≥8 h (*N* = 13). St. D, Standard deviation.

## 6. Discussion

The aim of the present study was to highlight the role of fatigue as one of the most important human factors in complex sociotechnical systems such as Vessel Traffic Services. Several subjective measures and an objective measure were used to study the intra- and inter-individual differences in fatigue in Vessel Traffic Operators, and their relationships with other personal and environmental variables: mental work load, work shifts, reaction time, and hours of usual sleep.

The study of fatigue, sleepiness, and mental work load in different work shifts (morning, afternoon, and night) showed an increase of the three variables in all the shifts, being the change considerably bigger during the night. This shift goes from 22:00 P.M. to 8:00 A.M., so it has a duration of 10 h: 3 hours more than the other shifts (morning 8–15; afternoon 15–22). The duration of the night shift, although it is in accordance with the legislation, goes against the recommendations given by the National Institute of occupational Health and Safety of the Spanish Ministry of Labor [(44), p. 455], which estates that night shifts should not be longer than morning shifts. It should be noted, also, that the operators do not have any scheduled rest periods, whereas the same document recommends introducing them and contemplating the benefits of taking a nap in night shifts before fatigue affects performance.

Regulations and recommendations for the correct organization of shifts vary depending on the country, but the impact of shift work and night work, both on health and on performance, is a global concern for governments and for workers (45), being the fatigue one of the key elements in this equation.

Our findings are in the same line as the indicated in massive studies, such as the Maastricht Cohort Study on Fatigue at Work, that concludes that “Shift work is associated with a higher prevalence of fatigue, especially in three-shift work” (46). We would add that research findings about this variable, especially in night shifts, should be seriously considered to make policies and to regulate work schedules.

The relation between occupational fatigue and safety has been widely studied in air and earth transportation, and, as a result, these sectors have developed a better regulation of shifts and required breaks. More studies and regulations are needed in other means of transport, such as the maritime or the rail transport, where there is an increasing interest in the effects of fatigue, as it has shown to be a key issue for staff, passengers, and goods safety (10, 47).

The results of the present study indicated also a significant positive correlation between fatigue and mental workload, but, although the TAC was designed with the aim of collecting objective measures of reaction time that could serve for our research purposes, we could not find significant relationships between these data and the other variables; also the differences in reaction time pre an post-work were no significant The fact that the data showed consistent increases in reaction time in Gijón, and not in Cartagena, together with the observational data collected in both sites can be explained attending to the different real work load in those two centers: whereas the Cartagena Operators have to control the traffic of around 43 ships per day, the average for the Gijón Operators is less than four ships per day (8.3% of the traffic in Cartagena). As data were collected in real working situations, they were affected by all the different events that occur during the work shifts. Thus, the operators’ duties interfered with the accomplishment of the task designed to measure the reaction time. We can conclude that this type of task is not the best tool to measure reaction time in real work settings, where attention has to be put in the job duties.

The findings about the importance of sleep are also relevant. The results of this study show that a higher number of hours of regular sleep reduces the increase in fatigue and mental workload experienced by operators throughout the shift. Sleeping enough hours (quantity of sleep) emerges as a protective factor against fatigue. Assessment of quality of sleep is also relevant in shift workers and no assessment was made of it in this study; future research should also take it into account, as disturbed sleep is, as well as lack of enough sleep, a common problem among shift workers (45). More research is needed on how to reduce the impact of shifts on poor sleep.

In conclusion, fatigue is a human element that can compromise the functioning of the complex sociotechnical system of Vessel Traffic Services. The findings of this field study show that there is a pressing need to regulate shifts and rest periods in order to reduce the increment of fatigue throughout the working hours, especially in the night shift, when the increase of fatigue is bigger. Quantity of sleep emerges as a protective factor against fatigue, so sleep hygiene programs that help to get enough hours of sleep should be implemented among shift workers, as they are in risk of being affected by lack of sleep.

## Data availability statement

The original contributions presented in the study are included in the article/supplementary material, further inquiries can be directed to the corresponding author.

## Ethics statement

The studies involving human participants were reviewed and approved by Technical University of Cartagena Ethics Committee. The patients/participants provided their written informed consent to participate in this study.

## Author contributions

FCM: conceptualization, software, data curation, writing—original draft, and investigation. VS-L: writing—review and editing. DM-T: writing—review and editing. JR-G: supervision, investigation, and methodology. JSM: supervision and data curation. CR: methodology and formal analysis. MP: methodology and formal analysis. IF: formal analysis and resources. LAD-S: writing—review and editing, and visualization. All authors contributed to the article and approved the submitted version.

## Conflict of interest

The authors declare that the research was conducted in the absence of any commercial or financial relationships that could be construed as a potential conflict of interest.

## Publisher’s note

All claims expressed in this article are solely those of the authors and do not necessarily represent those of their affiliated organizations, or those of the publisher, the editors and the reviewers. Any product that may be evaluated in this article, or claim that may be made by its manufacturer, is not guaranteed or endorsed by the publisher.
